# Effect of Third Molar Surgery on Sleep Health Parameters of Young Adults: An Observational Study

**DOI:** 10.3390/medicina60060858

**Published:** 2024-05-24

**Authors:** Ioulianos Apessos, Theodoros Lillis, Athanasios Voulgaris, Kostas Archontogeorgis, Paschalis Steiropoulos, Nikolaos Dabarakis

**Affiliations:** 1 Department of Dentoalveolar Surgery, Implantology and Oral Radiology, School of Dentistry, Faculty of Health Sciences, Aristotle University of Thessaloniki, 54124 Thessaloniki, Greece; theolillisdent@hotmail.com (T.L.); nikdab@gmail.com (N.D.); 2 Division of Dentistry, 424 General Military Training Hospital, 56429 Thessaloniki, Greece; 3 Department of Pneumonology, Medical School, Democritus University of Thrace, 68100 Alexandroupolis, Greece; thanasisvoul@hotmail.com (A.V.); k.archontogeorgis@yahoo.it (K.A.); steiropoulos@yahoo.com (P.S.)

**Keywords:** third molar surgery, oral surgery, surgical extraction, sleep quality, daytime sleepiness, insomnia severity, Pittsburgh Sleep Quality Index, Epworth Sleepiness Scale, Athens Insomnia Scale, pain perception

## Abstract

*Background and Objectives*: The role of surgical extraction of the third molar in patients’ sleep quality remains unclear, although it is one of the most common oral surgical procedures. The aim of this study is to assess the changes in patient-reported sleep health outcomes after third molar surgery and to investigate any associations between sleep parameters and post-extraction pain. *Materials and Methods*: Young adults without known comorbidities who were in need of mandibular third molar surgical extraction were included. All participants completed a sleep diary, the Epworth Sleepiness Scale (ESS), Pittsburgh Sleep Quality Index (PSQI) and Athens Insomnia Scale (AIS) questionnaires, which were used to assess sleep habits, daytime sleepiness, sleep quality and insomnia severity one week before and after extraction. In addition, a visual analog scale was completed postoperatively to assess the perception of pain. *Results*: Out of 75 patients who completed the study protocol, 32 (42.7%) were males and 43 (57.3%) were females, with a mean age of 24.01 (±3.43) years. Postoperatively, statistically significant higher scores were observed for PSQI [4.85 (±2.32) before vs. 5.39 (±2.75) after, *p* = 0.041], AIS [5.56 (±3.23) before vs. 6.91 (±4.06) after, *p* < 0.001] and average weekly number of nocturnal awakenings [2.01 (±3.72) before vs. 4.19 (±5.20) after, *p* < 0.001] but not for ESS, average weekly sleep duration and average weekly sleep onset latency. Pain perception was increased in patients who slept worse on almost all seven postoperative days, although this did not reach statistical significance. *Conclusions*: Third molar surgery impacts sleep quality and insomnia severity in the first week after extraction, while there is no effect on daytime sleepiness. The worsening of subjective sleep symptoms after extraction may be associated with an increased perception of pain.

## 1. Introduction

Third molars, commonly known as wisdom teeth, are the last teeth that erupt in the oral cavity. Surgical extraction of a third molar is one of the most common oral surgical procedures, which is usually performed on young adults who often have no previous experience with surgical procedures. It is usually performed between the ages of 20 and 29 [1]. The main indications for extraction are pericoronitis, caries of third molar and periodontal disease or caries of adjacent second molar [2]. 

Although surgical extraction of a third molar is a minor surgical procedure, it is characterized by post-extraction morbidity [3]. Pain and facial edema are common, while complications such as trismus, alveolar osteitis and paresthesia may also occur [4,5]. Oral-health-related quality of life is impaired, especially in the immediate postoperative period [6]. Controversy exists regarding subjectively reported postoperative sleep impairment [7,8,9]. Facial edema makes settling in bed more difficult, while pain may complicate both sleep onset and sleep maintenance.

Inadequate quantity and poor quality of sleep play a significant role in subjects’ health status [10]. Chronic insufficient sleep is associated with increased mortality risk and contributes to the societal burden associated with higher prevalence rates of cardiovascular disease, diabetes mellitus, obesity and cancer [11]. Insufficient sleep duration, irregular sleep timing and insomnia are common among young adults. Short-term sleep deprivation may impact physical health, mental health, mood and public safety [10]. These conditions often remain undiagnosed, as they may not impact the daily routine of individuals [12].

Sleep disturbance, early in the postoperative period, is common among surgical patients [13]. Polysomnographic studies on surgical patients reveal decreased total sleep time, loss of restorative sleep and sleep fragmentation [13,14]. Moreover, postoperative sleep disturbances can have harmful effects on postoperative recovery [15] and a reciprocal impact on pain [16]. 

Considering the high prevalence of sleep disorders and third molar impaction in young adults, it is important to assess the impact of third molar surgery on sleep health parameters. This assessment is crucial for making clinical decisions and giving adequate instructions during the postoperative period. Currently, there is limited evidence on the relationship between third molar surgery and sleep disorders. 

Thus, our aim was to investigate the changes in patient-reported sleep health outcomes, namely insomnia symptoms, daytime sleepiness and sleep quality, following third molar surgery compared to the pre-surgical sleep status. We further analyzed the association between post-extraction pain and changes in the aforementioned sleep parameters.

## 2. Materials and Methods

### 2.1. Study Design and Registration

This observational study was conducted at the School of Dentistry, Faculty of Health Sciences, Aristotle University of Thessaloniki, Greece. The study protocol was approved by the Ethics Committee of Dental School (165/16-06-2022), registered at the international Open Science Framework Registry (https://doi.org/10.17605/OSF.IO/EY9NF, accessed on 5 November 2023), and all procedures were conducted in accordance with the Helsinki Declaration of Human Rights [17]. Informed consent was provided by all patients upon enrollment in the study.

### 2.2. Study Population

Young adults (<30 years old) referred to the Department of Dentoalveolar Surgery for mandibular third molar surgical extraction from September 2022 to December 2023 were included. All participants were free from pain and had no acute symptoms related to the mandibular third molar. The inclusion criteria were as follows: good general health (ASA I) [18], pericoronitis or localized periodontitis as an indication for extraction and surgical difficulty assessed as “very difficult” based on the Pederson Index [19]. The exclusion criteria were as follows: history of systemic diseases, oral surgical interventions in the preceding two months, a previous diagnosis of sleep disorders and medication intake (either prescribed or off-label) that may affect sleep, including analgesic medication. After the initial clinical and radiographic examination, patients who met the above criteria were informed about the study and scheduled for the extraction appointment.

### 2.3. Clinical Procedures

Among patients who consented to participate in the study, sleep diaries were distributed to record the duration of their sleep and the latency for sleep onset seven days before and seven days after the extraction. The day of the appointment, before surgery, patients filled the following questionnaires: Epworth Sleepiness Scale (ESS), Pittsburgh Sleep Quality Index (PSQI) and Athens Insomnia Scale (AIS). All of them are validated questionnaires that are commonly used to assess daytime sleepiness, sleep quality and insomnia severity, respectively.

All extractions were performed under local anesthesia by third-year postgraduate students of the Master of Science Program in Oral Surgery. Operators were blinded to the sleep status of patients, while patients were not informed about their sleep status until the end of the study. The surgical extraction protocol included the prescription of Amoxicillin 2 gr (or Clarithromycin 500 mg, in case of allergy to penicillin) 30–60 min before extraction. The techniques of dental anesthesia and local anesthetics used were the same for all patients. Inferior alveolar nerve block anesthesia was performed using Mepivacaine 3%. Aspiration test preceded the injection of anesthetic solution in every case. Infiltration anesthesia, buccally, was performed using Lidocaine 2% with 1:80,000 epinephrine. A full-thickness mucoperiosteal envelope flap was prepared and raised, and then, ostectomy and odontomy were performed using a surgical handpiece and sterile saline irrigation. Elevators and forceps were used for tooth extraction. The extraction socket was irrigated with saline, and the flap was repositioned and sutured (3–0 silk). Niflumic acid 250 mg q.i.d. was prescribed for 2 days, and as needed afterward. Patients were given verbal and written post-extraction instructions. Finally, they were given the sleep diary and a visual analog scale (VAS) for pain perception related to the postoperative week and were guided how to fill them.

On the seventh day of follow-up, sutures were removed, wound healing was evaluated, and oral hygiene instructions were given. Patients returned the sleep diary and VAS and completed the ESS, PSQI and AIS questionnaires, related to the first postoperative week. 

### 2.4. Baseline and Follow-Up Measurements

#### 2.4.1. Daytime Sleepiness

Assessment of excessive daytime sleepiness was performed using the ESS questionnaire [20,21]. ESS also assesses the effectiveness of any treatment targeting the symptoms of daytime sleepiness [22]. It includes 8 questions pertaining to daily activities. Subjects were asked to rate the chance of dozing off or falling asleep in 8 different situations. The score for each question ranges from 0 to 3, and their sum is the final score for the questionnaire. Higher scores indicate a higher average sleep propensity [23]. An overall score in the ESS above 10 is suggestive of daytime sleepiness.

#### 2.4.2. Sleep Quality

PSQI is a standard self-assessment of sleep quality over the last month [24,25]. It consists of 19 questions grouped into 7 subcategories. The 7 distinct clinical subclasses of sleep difficulties are as follows: 1. Subjective sleep quality (one question), 2. Sleep latency (two questions), 3. Sleep duration (one question), 4. Sleep efficiency (three questions), 5. Sleep disorders (nine questions), 6. Use of sleep medication (one question), 7. Daytime dysfunction (two questions). These distinct subcategories are summed and produce an overall result, with a normal value of ≤5. Thus, subjects were divided into those with good (PSQI ≤ 5) and poor sleep quality (PSQI > 5).

#### 2.4.3. Insomnia Severity

AIS assesses the severity of insomnia using the diagnostic criteria according to the International Classification of Diseases-10 (ICD-10) [26]. The eight-item questionnaire evaluates sleep onset, night and early-morning waking, sleep time, sleep quality, frequency and duration of complaints, distress caused by insomnia and interference with daily functioning. Each item can be scored from 0 to 3 (with 0 corresponding to no problem and 3 to serious problem), and the total score ranges from 0 (absence of any problem related to sleep) to 24 (presence of the most severe degree of insomnia). A total score of 6 or higher is indicative of an insomnia diagnosis.

#### 2.4.4. Subjective Sleep Assessment

A custom two-week sleep diary was given to the participants, where they reported bedtime, the time they thought they fell asleep, the number and duration of nocturnal awakenings and the time they woke up in the morning. Sleep onset latency was calculated as the amount of time between the reported bedtime and fall asleep time. Sleep duration was calculated as the total amount of time between the reported sleep onset and wake-up time, minus the reported duration of nocturnal awakenings. Finally, the average weekly sleep duration and sleep onset latency were calculated for one week before and one week after third molar surgery.

#### 2.4.5. Pain Perception

For the week after the extraction, patients reported the daily intensity of pain based on a visual analog scale (VAS) ranging from 0 (did not feel any pain) to 10 (felt a lot of pain). Pain assessment was performed only after the procedure, as they had no complaints of pain before the extraction.

### 2.5. Sample Size Calculation

Sample size was calculated using G*Power 3.1 software. Our goal was to obtain 0.80 power to detect an effect size of 0.35 at the standard 0.05 alpha error probability. Our target minimum sample size was 70 participants. An attempt to recruit up to 90 participants was made, assuming that not all participants would complete the total task.

### 2.6. Statistical Analysis

For the statistical analysis of the data, descriptive statistical indices of central tendency and variability were calculated. A comparison of the PSQI score, ESS score, AIS score, average weekly sleep duration (in hours), average weekly sleep onset latency (in minutes) and the number of weekly nocturnal awakenings before and after surgical extraction was performed. The normality of the distribution of differences in the values was tested using the Kolmogorov–Smirnov normality test. A priori, the calculated sample was >50 participants. If the values were normally distributed, a paired sample *t*-test was conducted; otherwise, a Wilcoxon signed rank test was performed.

A subgroup analysis was conducted to investigate the possible associations between changes in sleep parameters and postoperative pain perception. Groups were divided on the basis of changes observed in PSQI, ESS or AIS scores, as well as in average weekly sleep duration (in hours), average weekly sleep onset latency (in minutes) and the number of weekly nocturnal awakenings. As a dependent variable, the VAS pain score of each postoperative day was used. The correlation between the variables was tested with Student’s *t*-test and Pearson’s correlation coefficient if the values were normally distributed; otherwise, the Mann–Whitney U-Test and Spearman’s rank correlation coefficient were used. 

All variables are presented as mean (±standard deviation) for continuous variables and absolute frequencies (relative frequencies %) for categorical variables. The statistical significance level of each test was set at 0.05. All analyses were carried out using SPSS IBM, v25.

## 3. Results

Out of 90 patients who were initially eligible for inclusion in the study, 15 were excluded because they either refused to complete the follow-up appointment (*n* = 5) or did not complete the scales or diaries that were given to them (*n* = 10), resulting in a sample of 75 patients who completed the whole study protocol. Of these, 32 (42.7%) were males and 43 (57.3%) were females. The mean age was 24.01 (±3.43) years; the mean BMI was 22.98 (±3.73) kg/m^2^; and the mean neck circumference was 36.27 (±4.28) cm (Table 1).

As expected, pain perception from Day 1 to Day 7 decreased gradually. The mean VAS pain score for Day 1 was 5.53 (±2.80); for Day 2, it was 4.70 (±2.59); for Day 3, it was 3.96 (±2.64); for Day 4, it was 3.37 (±2.71); for Day 5, it was 2.77 (±2.46); for Day 6, it was 1.88 (±2.24); and for Day 7, it was 1.35 (±1.92).

The mean values and comparisons before and after third molar surgery for ESS, PSQI and AIS scores, as well as for average weekly sleep duration (in hours), average weekly sleep onset latency (in minutes) and weekly number of nocturnal awakenings, are presented in Table 2. Preoperatively, based on the questionnaires, 27 patients had poor sleep quality, 39 subjectively reported symptoms of insomnia and 12 of excessive daytime sleepiness. Postoperatively, 28 patients had poor sleep quality, 44 subjectively reported symptoms of insomnia and 11 of excessive daytime sleepiness. Significant changes were observed for PSQI scores (*p* = 0.041), AIS scores (*p* < 0.001) and the weekly number of awakenings (*p* < 0.001) but not for the ESS score (*p* = 0.707), average weekly sleep duration (*p* = 0.648) and average weekly sleep onset latency (*p* = 0.074).

As the next step, groups were divided based on the changes (worse vs. same or better subjective sleep scores) observed in PSQI, ESS or AIS scores, as well as in average weekly sleep duration (in hours), average weekly sleep onset latency (in minutes) and the number of weekly nocturnal awakenings. The first week after surgical extraction, the ESS score increased in 24 (32%) patients, the PSQI score increased in 34 (45.3%) patients, and the AIS score increased in 37 (49.3%) patients. Increased sleep onset latency was observed in 40 (53.3%) patients, increased number of nocturnal awakenings in 47 (62.7%) patients and decreased sleep duration in 31 (41.3%) patients. 

In the subgroup analysis, a significant difference between increased and decreased sleep onset latency was demonstrated only in the VAS pain score on the first post-extraction day (*p* = 0.011). A positive correlation was observed between sleep onset latency change and VAS pain score on Day 1 (rho = 0.286, *p* = 0.013), between daytime sleepiness change and VAS pain score on Day 5 (rho = 0.229, *p* = 0.048) and between the number of nocturnal awakenings change and VAS pain score on Day 6 (rho = 0.228, *p* = 0.049).

**Figure 1:**
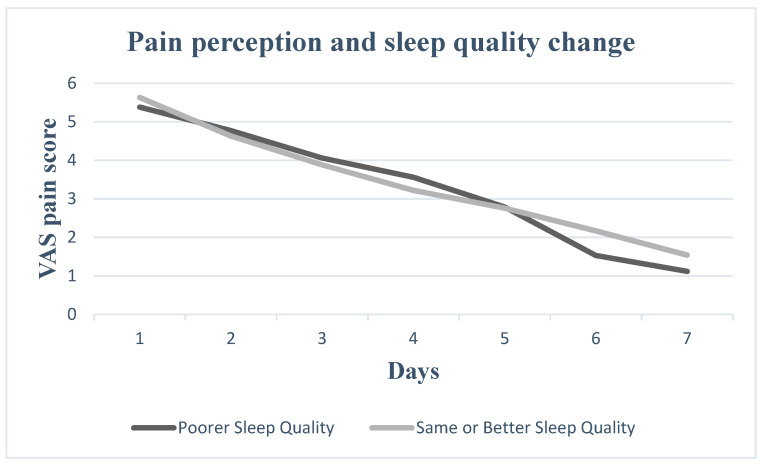
VAS pain score for the first seven post-extraction days in patients with and without sleep quality change.

**Figure 2:**
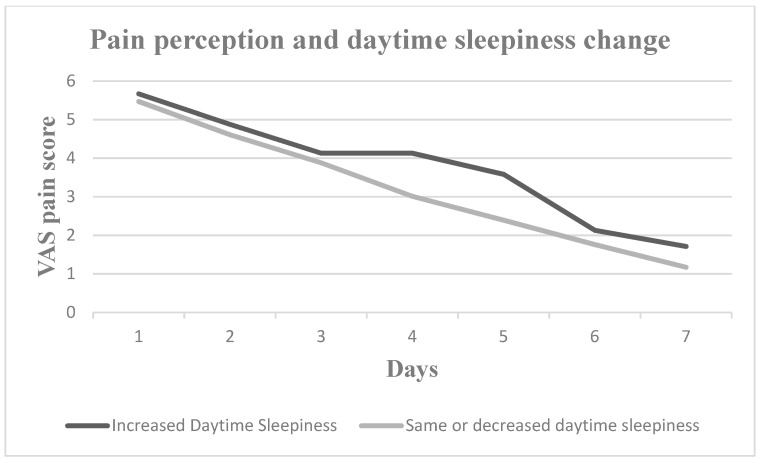
VAS pain score for the first seven post-extraction days in patients with and without daytime sleepiness change.

**Figure 3:**
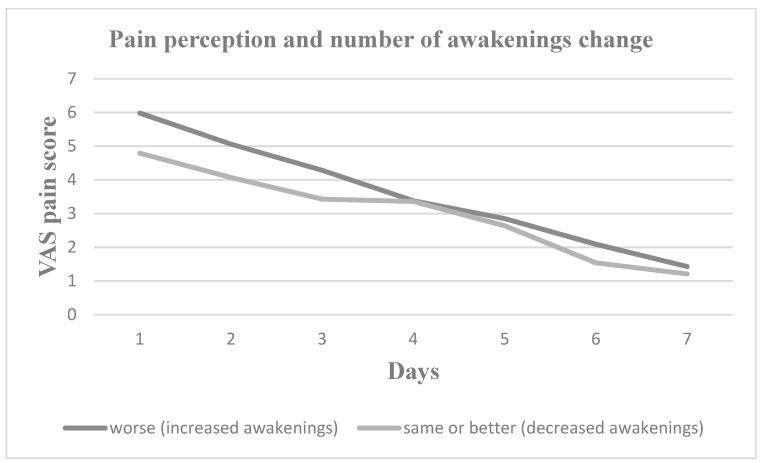
VAS pain score for the first seven post-extraction days in patients with and without changes in the number of nocturnal awakenings.

**Figure 4:**
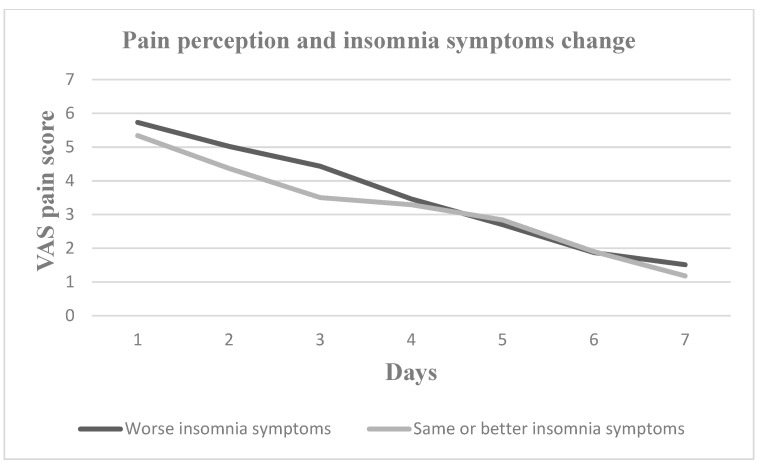
VAS pain score for the first seven post-extraction days in patients with and without insomnia symptoms change.

**Figure 5:**
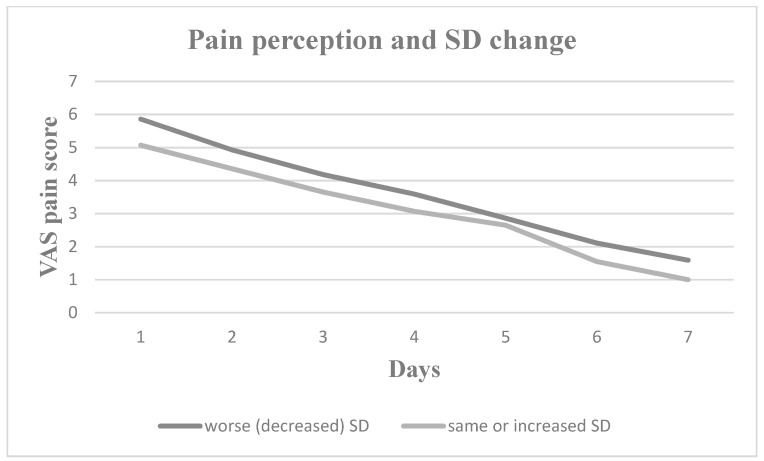
VAS pain score for the first seven post extraction days in patients with and without sleep duration (SD) change.

**Figure 6:**
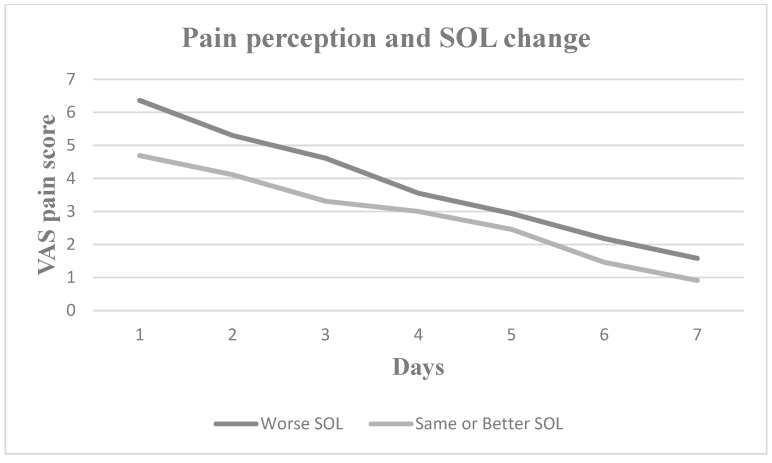
VAS pain score for the first seven post extraction days in patients with and without sleep onset latency (SOL) change.

Nevertheless, pain perception increased for all seven postoperative days in patients who experienced increased daytime sleepiness, decreased sleep duration, increased sleep onset latency and increased number of nocturnal awakenings. Patients with worse sleep quality experienced greater pain on Days 2, 3, 4 and 5, whereas patients with more severe subjective insomnia symptoms experienced greater pain on Days 1, 2, 3, 4 and 7. In Figure 1, Figure 2, Figure 3, Figure 4, Figure 5 and Figure 6, the trajectory of VAS pain score from Day 1 to 7 is presented regarding changes in the investigated sleep health parameters.

## 4. Discussion

The present study demonstrated that mandibular third molar surgery influences sleep quality and subjective symptoms of insomnia in the first week after extraction. Sleep fragmentation occurs due to the higher number of nocturnal awakenings. However, there was no impact on daytime sleepiness, sleep duration and sleep onset latency. Although patients reporting pain at a greater degree had a more disturbed sleep architecture than those with less pain, the difference was not statistically significant for most of the studied sleep variables. To the best of our knowledge, this is the first study that investigates the effect of third molar surgery on sleep health parameters.

Sleep disorders are commonly encountered over a person’s life course, even in those who are otherwise healthy. Previous studies estimate the prevalence of insomnia at approximately 30% and of insufficient sleep duration at 32.5% in the adult population [10,27], while recent evidence estimates that 936 million adults worldwide suffer from mild-to-severe OSA [28]. Importantly, sleep disorders have a significant impact on a person’s well-being and are also responsible for the development and worsening of several medical conditions [29]. All of the above highlights the need for and importance of health professionals’ awareness regarding both the recognition of sleep disorders and interventions that induce them. 

A population-based study reported that approximately half of adults undergo at least one third molar extraction by the age of 25 years [30]. Scientific dental societies’ special interest is focused on the necessity for prophylactic extraction and the financial burden of insurance systems either from an asymptomatic tooth surgical extraction or a watchful monitoring strategy [2,31,32]. Third molar surgery is considered an issue with public health implications. In fact, for the accretive incidence of sleep disorders, perioperative sleep disturbances is a parameter that should not be overlooked [11].

Our study showed that third molar surgery may impose a risk on sleep health, even for a short period of time, with a postoperative deterioration in both sleep quality and insomnia severity symptoms. These findings can be attributed to pain, changes in sleep habits due to awakening for analgesic drug consumption and discomfort due to edema and trismus. Social distancing, eating and speaking difficulties are present for a few days, changing the daily routine, which inevitably affects the sleep–wake cycle. Of note, inflammatory cytokines that are released in response to surgery, such as IL-1, IL-6 and TNF, are related to disrupted sleep [13].

It has been widely accepted that sleep and pain are related, whereas recent data suggest that sleep disturbance may be a stronger predictor of pain than pain is for sleep disturbance [33]. Sleep deprivation can induce hyperalgesic responses in healthy individuals, which can be reversed by short sleep [34]. On the other hand, adult patients who experienced pain had worse measures of sleep onset latency, time awake after onset and recurrent awakenings [35]. In our study, patients with worsening sleep parameters after extraction felt greater pain. Conversely, in the subgroup analysis, we found no statistically significant difference for most of the studied subjective sleep variables as far as pain perception at the first post-extraction week was concerned. This was reasonable because patients were under treatment with niflumic acid every six hours for the first two postoperative days, and pro re nata afterward.

Several studies have previously examined the impact of third molar surgery on the quality of life using the Oral Health Impact Profile-14 (OHIP-14) or custom self-administered scales [6]. Oral-health-related quality of life was impaired for the first seven days after extraction, and this was attributed to pain, edema and trismus. Sleep impairment was investigated using questions related to subjective sleep onset latency, sleep duration, nocturnal awakenings and total duration of sleep disturbances. Braimah RO et al. reported that sleep was affected in 37.8% of patients on the first postoperative day (POD). Ibikunle AA et al. found that patients who did not receive prednisolone before third molar surgery reported sleep impairment in a higher percentage compared with patients who received prednisolone orally or submucosally, and the difference was statistically significant in the first and third POD. Starch-Jensen T et al. recorded approximately 30% of patients with problems falling asleep, interruptions during sleep and drowsiness after extraction, while Colorado-Bonnin M. et al. applied the same questions and reported a percentage of approximately 45%. Interestingly, no study has used validated scales designed for screening sleep disorders [7,9,36]. The present study used three scales for the screening of different aspects of sleep health, namely ESS for daytime sleepiness, PSQI for sleep quality and AIS for insomnia severity. All three scales are validated in the Greek language. In this way, the results are comparable both to studies coming from different countries investigating the same issue and to studies investigating the effect of other surgical interventions on sleep. 

Dentists and oral surgeons should be aware of postoperative sleep quality deterioration and insomnia incidence, and they should realize the burden of sleep disorders; thus, they should incorporate some questions related to sleep into their history taking and provide sleep hygiene guidance along with postsurgical instructions. Nocturnal awakenings may be limited if the dosage of analgesic drugs is adapted properly in order to allow at least seven hours of “painless” sleep. Another matter that should be considered is driving capability, which may be impaired, increasing the risk of accidents and injuries caused by sleepiness and fatigue, including workplace accidents and motor vehicle crashes [37,38]. A couple of days off work or short naps during work breaks are recommended [39].

The present study is subject to several limitations, namely the lack of a control group and the fact that it is a questionnaire-based study. Thus, the results should be interpreted with caution. Sleep duration, nocturnal awakenings and sleep onset latency are assessed subjectively by patients through sleep diaries and not using objective methods, such as multiple sleep latency test, multiple wakefulness test and polysomnography. Moreover, the sample size is relatively small; therefore, the findings cannot be extrapolated to the general population. In addition, other factors might have influenced the postoperative sleep of the included patients, such as their pre-existing mental status, the duration and recovery from the surgical procedure and the use of analgesic medication to properly control pain, among others. Finally, the extractions were performed by oral surgery senior residents—a fact that might have influenced our results, as more experience is associated with less postsurgical discomfort [40].

## 5. Conclusions

In conclusion, during the first week after extraction of a third molar, sleep quality and insomnia severity are affected, whereas the number of nocturnal awakenings is increased. On the other hand, no effect of surgical extraction on daytime sleepiness, sleep onset latency and sleep duration for the first postoperative week is observed. Pain perception after extraction is increased in patients, accompanied by the worsening of subjective sleep symptoms. Further research is necessary, however, to elucidate the interaction between third molar extraction and sleep characteristics.

## Figures and Tables

**Table 1:** Anthropometric measurements and demographics.

Variable	Measurement
Gender (male/female)	32/43
Age (mean ± SD)	24.01 ± 3.43 years
BMI (mean ± SD)	22.98 ± 3.73 kg/m^2^
Neck circumference (mean ± SD)	36.27 ± 4.28 cm

Abbreviations: BMI: Body Mass Index, SD: Standard Deviation.

**Table 2:** Comparison of scores of sleep questionnaires and reported sleep characteristics before/after the surgery.

Measurement	Before the Third Molar Surgery	After the Third Molar Surgery	*p*
ESS score	6.32 (±3.99)	6.48 (±3.85)	0.707
PSQI score	4.85 (±2.32)	5.39 (±2.75)	0.041
AIS score	5.56 (±3.23)	6.91 (±4.06)	<0.001
Average weekly sleep duration (h)	7.36 (±1.15)	7.41 (±1.16)	0.648
Average weekly sleep onset latency (min)	25.10 (±21.66)	26.33 (±19.27)	0.074
Weekly nocturnal awakenings	2.01 (±3.72)	4.19 (±5.20)	<0.001

Abbreviations: ESS: Epworth Sleepiness Scale, PSQI: Pittsburgh Sleep Quality Index, AIS: Athens Insomnia Scale.

## Data Availability

Data available on request from the authors.
